# Fatal meningoencephalitis associated with Ebola virus persistence in two survivors of Ebola virus disease in the Democratic Republic of the Congo: a case report study

**DOI:** 10.1016/S2666-5247(24)00137-X

**Published:** 2024-10

**Authors:** Daniel Mukadi-Bamuleka, François Edidi-Atani, Maria E Morales-Betoulle, Anaïs Legand, Antoine Nkuba-Ndaye, Junior Bulabula-Penge, Placide Mbala-Kingebeni, Ian Crozier, Fabrice Mambu-Mbika, Shannon Whitmer, Olivier Tshiani Mbaya, Lisa E Hensley, Richard Kitenge-Omasumbu, Richard Davey, Sabue Mulangu, Peter N Fonjungo, Michael R Wiley, John D Klena, Martine Peeters, Eric Delaporte, Johan van Griensven, Kevin K Ariën, Catherine Pratt, Joel M Montgomery, Pierre Formenty, Jean-Jacques Muyembe-Tamfum, Steve Ahuka-Mundeke, EBOV Persistence Study Group, Anja De Weggheleire, Gnakub N Soke, Raymond Pallawo, Gouressy Ibrahima, Victor Epaso Gelege, John Kombe-Ngwama, Grace Kahambwe-Ekoko, Mathias Mossoko Gbe, Pierre-Céleste Adikey Limne, Etienne Yuma-Kibondo, Eddy Kinganda-Lusamaki, Adrienne Amuri Aziza, Yannick Tutu Tshia N'kasar, Elias Mumbere Kalemekwa, Divine Kitsa-Mutsumbirwa, Noella Mulopo-Mukanya, Fyfy Mbelu-Matulu, Marie-Anne Kavira-Muhindo, Jacques Kwizera Sendegeya, Hugo Kavunga-Membo

**Affiliations:** a Department of Virology, Institut National de Recherche Biomédicale, Kinshasa, Democratic Republic of the Congo; b Service of Microbiology, Department of Medical Biology, Kinshasa University Hospital, University of Kinshasa, Kinshasa, Democratic Republic of the Congo; c Rodolphe Mérieux Institut National de Recherche Biomédicale–Goma Laboratory, Goma, Democratic Republic of the Congo; d US Centers for Disease Control and Prevention, Atlanta, GA, USA; e Health Emergencies Programme, WHO, Geneva, Switzerland; f Clinical Monitoring Program Research Directorate, Frederick National Laboratory for Cancer Research, Frederick, MD, USA; g Zoonotic and Emerging Disease Research Unit, US Department of Agriculture National Bio and Agro-Defense Facility, Manhattan, KS, USA; h National Institute for Allergy and Infectious Diseases–National Institutes of Health, Rockville, MD, USA; i Programme Nationale d’Urgences et Actions Humanitaires, Ministry of Health, Kinshasa, Democratic Republic of the Congo; j University of Nebraska Medical Center, Omaha, NE, USA; k PraesensBio, Omaha, NE, USA; l TransVIHMI, Université de Montpellier–IRD–INSERM, Montpellier, France; m Department of Clinical Sciences, Institute of Tropical Medicine Antwerp, Antwerp, Belgium; n Department of Biomedical Sciences, Institute of Tropical Medicine Antwerp, Antwerp, Belgium; o Department of Biomedical Sciences, University of Antwerp, Antwerp, Belgium; p Biosurv International, Salisbury, UK

## Abstract

**Background:**

During the 2018–20 Ebola virus disease outbreak in the Democratic Republic of the Congo, thousands of patients received unprecedented vaccination, monoclonal antibody (mAb) therapy, or both, leading to a large number of survivors. We aimed to report the clinical, virological, viral genomic, and immunological features of two previously vaccinated and mAb-treated survivors of Ebola virus disease in the Democratic Republic of the Congo who developed second episodes of disease months after initial discharge, ultimately complicated by fatal meningoencephalitis associated with viral persistence.

**Methods:**

In this case report study, we describe the presentation, management, and subsequent investigations of two patients who developed recrudescent Ebola virus disease and subsequent fatal meningoencephalitis. We obtained data from epidemiological databases, Ebola treatment units, survivor programme databases, laboratory datasets, and hospital records. Following national protocols established during the 2018–20 outbreak in the Democratic Republic of the Congo, blood, plasma, and cerebrospinal fluid (CSF) samples were collected during the first and second episodes of Ebola virus disease from both individuals and were analysed by molecular (quantitative RT-PCR and next-generation sequencing) and serological (IgG and IgM ELISA and Luminex assays) techniques.

**Findings:**

The total time between the end of the first Ebola virus episode and the onset of the second episode was 342 days for patient 1 and 137 days for patient 2. In both patients, Ebola virus RNA was detected in blood and CSF samples during the second episode of disease. Complete genomes from CSF samples from this relapse episode showed phylogenetic relatedness to the genome sequenced from blood samples collected from the initial infection, confirming in-host persistence of Ebola virus. Serological analysis showed an antigen-specific humoral response with typical IgM and IgG kinetics in patient 1, but an absence of an endogenous adaptive immune response in patient 2.

**Interpretation:**

We report the first two cases of fatal meningoencephalitis associated with Ebola virus persistence in two survivors of Ebola virus disease who had received vaccination and mAb-based treatment in the Democratic Republic of the Congo. Our findings highlight the importance of long-term monitoring of survivors, including continued clinical, virological, and immunological profiling, as well as the urgent need for novel therapeutic strategies to prevent and mitigate the individual and public health consequences of Ebola virus persistence.

**Funding:**

Ministry of Health of the Democratic Republic of the Congo, Institut National de Recherche Biomédicale, Infectious Disease Rapid Response Reserve Fund, US Centers for Disease Control and Prevention, US National Cancer Institute (National Institutes of Health), French National Research Institute for Development, and WHO.

## Introduction

Since the discovery of Ebola virus in 1976, the Democratic Republic of the Congo has experienced 15 Ebola virus disease outbreaks, including the second largest in history from Aug 1, 2018, to June 25, 2020, which involved 3470 confirmed and probable cases and 2287 deaths.^1^ The public health response was notable, with widespread use of specific therapeutics and vaccination, as well as improved supportive care; 1044 patients with confirmed Ebola virus disease received vaccination or experimental therapeutics, likely resulting in improved survival.^2^, ^3^, ^4^

**Research in context**

**Evidence before this study**

Until the past decade, the number of survivors of Ebola virus disease was historically low, and their risk of clinical sequelae following the acute stage of disease, as well as viral persistence, was poorly characterised. After the devastating 2014–16 outbreak of Ebola virus disease in western Africa, the persistence of Ebola virus in immune-privileged sites associated with either severe clinical syndromes or a risk of human-to-human transmission was first documented. We searched PubMed, without language restrictions, for articles published from Sept 1, 1976, to Nov 30, 2023, using the following terms: “Ebola virus disease,” “Ebola virus,” “meningitis,” “meningoencephalitis,” and/or “relapse,” and/or “recrudescence”. We found 77 papers, including descriptions of neurological manifestations associated with Ebola virus disease (seven papers) and of Ebola virus persistence and disease relapse or recrudescence (nine papers). Only one study definitively associated the detection of infectious Ebola virus in cerebrospinal fluid (CSF) with meningoencephalitis in a survivor of Ebola virus disease from the UK, implicating the CNS as a possible site for viral persistence. Work conducted in non-human primate models also documented long-term persistence of Ebola virus in the CNS of animals who had received treatment, raising questions about viral persistence in the CNS of human survivors after receiving therapeutics specific to Ebola virus.

**Added value of this study**

After the 2018–20 outbreak of Ebola virus disease in the Democratic Republic of the Congo, a national survivor programme enabled follow-up, care, and documentation of events that might have been missed in the past. Although the UK case provided the first proof-of-principle study, our findings add novel and unique insights and important updates. This case study reports the first high-resolution cases of meningoencephalitis associated with persistence of Ebola virus in the CNS in two survivors of Ebola virus disease in an African setting (in which all known outbreaks have originated). Notably, these cases emerged in the context of a new standard of care, in which vaccines and monoclonal antibody (mAb) therapeutics specific to Ebola virus have been routinely deployed to almost all close contacts or patients. Furthermore, we describe the first report of viral persistence in the CNS associated with an absence of endogenous adaptive humoral immune responses in a patient who had received vaccination and a mAb therapeutic. We provide detailed insight into the clinical course, including vaccination and treatment history, of two patients with Ebola virus disease who developed fatal meningoencephalitis months into their convalescence. A combined analysis of samples from the initial and relapse episodes by use of molecular testing (quantitative RT-PCR), complete genome sequencing, and serological assays (IgM and IgG ELISA), including a novel investigation of antibodies in the CSF, provides insight into the pathogenesis of Ebola virus disease. A genomic analysis allowed us to rule out selection of virus mutants that escaped treatment with mAbs in the CNS.

**Implications of all the available evidence**

Many survivors of Ebola virus disease living in the Democratic Republic of the Congo have received vaccines and therapeutics specific to Ebola virus and are potentially at risk of viral persistence and its consequences. Our findings highlight the importance of long-term clinical, virological, and immunological monitoring for all survivors and enhanced surveillance in areas where they reside. The reported events occurred in remote areas of the Democratic Republic of the Congo; we provide detailed descriptions of each case to increase the awareness of health-care providers of possible viral persistence in the CNS as a cause of meningoencephalitis in individuals residing in areas affected by Ebola virus, survivors of Ebola virus disease, or both. Additionally, our results highlight the need to develop therapeutic strategies that can prevent or reduce viral persistence in the CNS; to conduct in-depth, longitudinal, immunological studies of survivors after receiving vaccination, approved therapeutics, or both during outbreaks; and to further characterise, understand, and identify biomarkers of viral persistence in the CNS to improve individual and public health outcomes.

Ebola virus can persist in the body fluids of survivors of Ebola virus disease, including semen, breast milk, aqueous humour, and cerebrospinal fluid (CSF), and persistence has been associated with meningoencephalitis, uveitis, and relapse of Ebola virus disease-like disease. Immune-privileged tissues, including the testes and CNS, are hypothesised to serve as sources of Ebola virus persistence.^5^, ^6^, ^7^, ^8^, ^9^, ^10^, ^11^

Encephalitis and meningoencephalitis have been reported during acute Ebola virus disease and convalescence.^12^, ^13^, ^14^, ^15^, ^16^ Additionally, among three well documented cases of Ebola virus disease relapse, one patient developed severe meningoencephalitis 9 months after discharge.^14^ Based on viral genome sequence analysis of samples taken from different anatomical sites after comparing acute-to-relapse infection, the CNS was hypothesised to be the likely source of persistent infection and recrudescent meningoencephalitis; no interference with the patient’s anamnestic humoral immune response to Ebola virus was described.^14^ Ebola virus has also been detected in the CNS in non-human primate models of Ebola virus disease.^17^^,^^18^ In primate models infected with Ebola virus and treated with monoclonal antibodies (mAbs), Ebola virus RNA persistence in the brain ventricular system was associated with severe clinical disease and pathology.^18^^,^^19^ It has not been established whether this was a generic consequence of effective rescue from an otherwise lethal disease or specifically related to the use of mAb therapeutics that might not penetrate the CNS.

We aimed to report the cases of two previously vaccinated and mAb-treated survivors of Ebola virus disease who developed a second episode of Ebola virus disease months after initial discharge, subsequently manifesting as fatal meningoencephalitis associated with the detection of Ebola virus RNA in CSF samples. In both cases, we provide detailed information on the disease timeline; clinical, laboratory, and serological features; and genomic sequencing. Additionally, we discuss the clinical implications for survivors of Ebola virus disease.

## Methods

### Study design and patients

In this case report study, we describe the presentation, management, and subsequent investigations of two patients who developed recrudescent Ebola virus disease after initial survival. Both events occurred towards the end of the 2018–20 outbreak of Ebola virus disease in the Democratic Republic of the Congo in remote areas of the North Kivu province, where the antiviral drug remdesivir was not immediately available. Retreatment with a mAb-based therapeutic was not provided to either patient given the clinical presentation.

For both patients, we obtained data from epidemiological databases, Ebola treatment units (ETUs), survivor programme databases, laboratory datasets, and hospital records. These data comprised the dates of potential exposure to Ebola virus disease, vaccination with r-VSV-ZEBOV-GP (Ervebo; Merck, Kenilworth, NJ, USA), admission to an ETU, treatment with REGN-EB3 (Inmazeb; Regeneron Pharmaceuticals, Tarrytown, NY, USA) during the first episode of Ebola virus disease, and hospital discharge (appendix 2 p 8). Additionally, we collected clinical information recorded during relapse, including the development of meningoencephalitis and fatal outcomes, and any laboratory analysis and differential diagnostics performed.

The study was approved by the Secretariat Technique du Comité Multisectoriel de lutte contre la Maladie à Virus Ebola and the Ministry of Health of the Democratic Republic of the Congo. Oral consent was obtained from each patient before ETU admission and sample collection. Clinical presentation, investigations, and care were provided as part of response protocols for the Ebola virus disease outbreak. All data presented here were de-identified with careful attention to maintain patient confidentiality at all times.

### Procedures

Blood, plasma, and CSF samples were collected according to survivor programme and Ebola virus disease response protocols established in the Democratic Republic of the Congo. Samples from each patient during the first and second episodes of disease were inactivated and tested by quantitative RT-PCR (RT-qPCR) with the GeneXpert Ebola Assay (Cepheid, Sunnyvale, CA, USA) to detect Ebola virus RNA targeting the genes encoding nucleoprotein (NP) and glycoprotein (GP).

RNA was extracted from different specimens, including blood, plasma, and CSF, and sequenced with a targeted enrichment or amplicon-based technique on the Illumina platform MiSeq (Illumina, San Diego, CA, USA). We performed a Bayesian phylodynamic analysis to construct a time-resolved phylogenetic tree, which included 677 genomes from patients with confirmed Ebola virus from the North Kivu and Ituri regions that were obtained by sequencing throughout the outbreak. Most of these sequences were obtained throughout the outbreak and publicly available at the time of this study; a few additional sequences were generated during this work. This analysis was shared on Nextstrain and GitHub (appendix 3). We also performed a molecular clock rate analysis to establish if viral RNA from the samples of patients with persistent infection showed a delayed substitution rate (appendix 2 pp 4–5).

We performed the serological detection of anti-Ebola virus IgM and IgG antibodies using the US Centers for Disease Control and Prevention (CDC)’s in-house ELISA with inactivated Ebola virus antigens (Ebola virus, Mayinga strain; species *Orthoebolavirus zairense*). All assays included known positive and negative controls, which samples were compared with following previously described protocols (appendix 2 pp 5–6). We performed a Luminex assay with four commercially available recombinant Ebola virus proteins from *O zairense* to evaluate anti-Ebola virus IgG antibodies on x.MAP Technology (Luminex, Austin, TX, USA; appendix 2 pp 6–7). These proteins were NP, GP (Kissidougou and Mayinga strains), and viral protein 40 (VP40). Additional details on the serological methods used can be found in appendix 2 (pp 5–7).

### Role of the funding source

The funders of the study had no role in study design, data collection, data analysis, data interpretation, or writing of the report.

## Results

**Figure 1:**
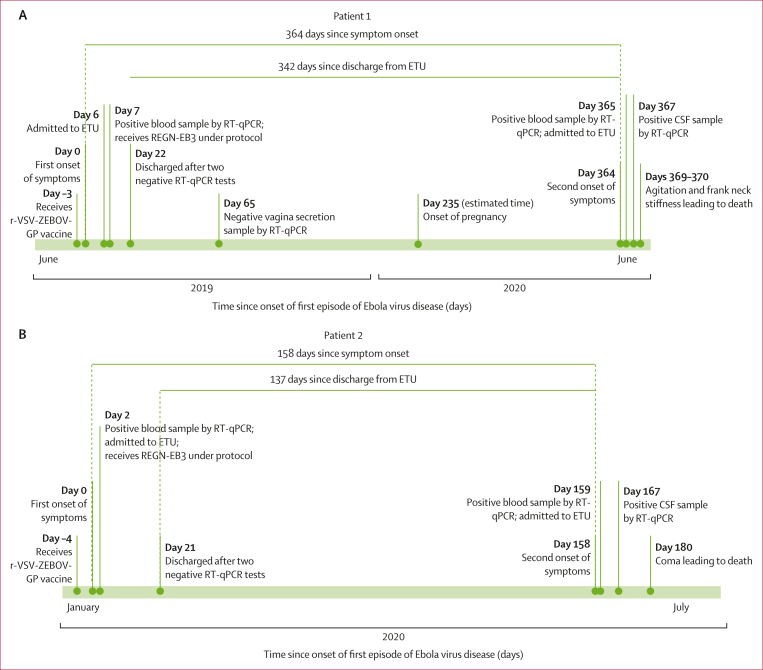
Timeline of Ebola virus disease progression in two patients Timeline of events from the first and second episodes of Ebola virus disease for patient 1 from June, 2019, to June, 2020 (A) and for patient 2 from January to July, 2020 (B). CSF=cerebrospinal fluid. ETU=Ebola treatment unit. RT-qPCR=quantitative RT-PCR.

A woman aged 22 years presented with nausea, asthenia, loss of appetite, and headaches that started in June, 2019 (patient 1). She consulted a communal health clinic (located in a remote area of North Kivu), where she was reportedly in contact with two patients with confirmed Ebola virus disease. 2 days later, she was referred to an ETU but repeatedly tested negative for Ebola virus by RT-qPCR. 5 days later, she was subsequently discharged from the ETU and received the r-VSV-ZEBOV-GP vaccine (figure 1A). 3 days after discharge, she presented with fever, headache, diarrhoea, and asthenia; 2 days later, she developed abdominal pain, arthralgia, and myalgia. On the following day, she consulted the local health centre, which raised the alert. Due to security concerns in the area and difficulty of transportation, she was unable to be transferred to an ETU until 3 days later, where Ebola virus disease was confirmed by RT-qPCR (cycle threshold [Ct] values: 27·4 for NP and 30·6 GP). The following day, she was treated with the REGN-EB3 mAb cocktail (150 mg/kg intravenously) and supportive care.^3^^,^^4^ Ebola virus RNA became undetectable 20 days after symptom onset and she was discharged following sequential negative RT-qPCR tests. Over the next 10 months, she attended monthly consultations provided by the programme for survivors of Ebola virus disease, where persistent visual disturbances, bulimia, and headaches were documented. 7 months after initial discharge, she reported being pregnant. 11 months after ETU discharge, she reported acute anorexia, nausea, asthenia, headache, and cough, and was clinically diagnosed with malaria. The following day, malaria testing was negative, but Ebola virus RNA was detected in a blood sample by RT-qPCR (Ct values: 33·6 for NP and 35·1 for GP). She was admitted to the ETU with a second episode of Ebola virus disease (figure 1A; appendix 2 p 8). Rapid diagnostic tests for syphilis, anti-HIV antibodies, and anti-hepatitis C virus antibodies were negative. Clinical treatment included artesunate, ceftriaxone, gentamicin, and paracetamol; she did not receive additional therapy specific to Ebola virus. After admission, Ebola virus RNA was detected at decreasing concentrations in daily blood samples that were also tested for clinical laboratory parameters until day 4 of the second episode (appendix 2 pp 11–12). Clinical manifestations reported at admission persisted, and new-onset fever and neck stiffness led to a presumptive diagnosis of meningoencephalitis. 3 days following the onset of these symptoms, Ebola virus RNA was detected in the CSF at a much higher concentration than in the blood (appendix 2 p 9). Over the next 2 days, the patient progressively deteriorated, marked by agitation and increased neck stiffness, and died 5 days after the onset of the second episode. Safe and dignified burial was conducted without fetus separation with family agreement.

A man aged 79 years (patient 2), a known contact of several patients with confirmed Ebola virus disease, closely cared for a family member with confirmed Ebola virus disease and participated in his burial in January, 2020. He received the r-VSV-ZEBOV-GP vaccine the following day, 1–9 days after presumptive exposure to Ebola virus (figure 1B; appendix 2 p 8). 4 days after vaccination, he developed anorexia (first day of illness, referred to as day 0); then asthenia (day 1 after symptom onset); followed by abdominal pain, myalgia, and polyarthralgia (day 2). He was referred to an ETU, where Ebola virus disease was confirmed by RT-qPCR (Ct values: 25·0 for NP and 29·7 for GP) on the third day of illness (day 2). Initial treatment included REGN-EB3 (150 mg/kg intravenously) and supportive care.^3^^,^^4^ New-onset dyspnoea with low oxygen saturation required oxygen therapy for 14 days; electrolyte imbalance was corrected; and the patient received corticosteroid therapy (indication, duration, and dosing was inconsistently reported in medical records). After progressive clinical improvement and complete clearance of Ebola virus RNA 21 days after symptom onset, he was discharged from the ETU following sequential negative RT-qPCR tests. Over the next 4–5 months, he attended monthly consultations at the Ebola virus disease survivor programme, reporting intermittent arthralgia, anorexia, and intense fatigue, but no neurological symptoms. 5 months after the first episode of Ebola virus disease, he presented with headache, anorexia, asthenia, abdominal pain, myalgia, arthralgia, and cough. Ebola virus RNA was detected in a blood sample by RT-qPCR, and the patient was transferred to the ETU on the same day having been diagnosed with the second episode of disease (appendix 2 pp 8, 10). Supportive care included artesunate, ceftriaxone, gentamicin, and intravenous volume repletion; he did not receive additional therapy specific to Ebola virus. On day 6 of the second episode, Ebola virus RNA was undetectable in his blood and the patient was transferred to a health-care facility for ongoing care needs. Subsequent clinical deterioration included agitation and altered mental status (spatial and temporal disorientation). After clinical staff suspected acute meningoencephalitis, Ebola virus RNA was detected in CSF (Ct values: 28·4 for NP and 32·6 for GP), but not in a coincident blood sample on day 9 of the second episode. Rapid diagnostic tests for syphilis, anti-HIV antibodies, and anti-hepatitis C virus antibodies were negative. On referral to another health-care facility, clinical examination noted typical vital signs, bedridden status, cognitive impairment, inability to speak (mutism or dysarthria), and four-limb hypotonia. Supportive care with ceftriaxone, quinine, phenobarbital, and dexamethasone was continued with no improvement. Subsequent clinical deterioration to coma occurred; severe hyponatraemia and a markedly elevated concentration of C-reactive protein were noted (appendix 2 p 13). Ebola virus RNA was still detected in CSF (Ct values: 34·4 for NP and 37·4 for GP) on day 21 of the second episode. The patient died on day 22 of the second episode.

To establish whether the second episodes of Ebola virus disease represented new infections or relapses of the previous infections, we sequenced residual blood and CSF samples taken from both patients during the second episode (patient 1: 20FHV038 [blood] and 20FHV036 [CSF]; patient 2: 20FHV039 [blood] and 20FHV037 [CSF]). For comparison, we sequenced samples taken during the first episode of Ebola virus disease for both patients (patient 1: MAN5036 [blood]; patient 2: MAN14228 [plasma]; appendix 2 pp 9–10).

For each patient, we generated complete genomes from blood (patient 1) or plasma (patient 2) samples collected during the first episode of Ebola virus disease and CSF samples collected during the second episode. For patient 2, using blood samples collected during the second episode, we also obtained a nearly complete genome that was identical to the genome from the CSF sample taken on the same day. For patient 1, both blood and CSF samples were sequenced; however, CSF had full coverage, whereas blood only had partial coverage (appendix 2 p 9).

**Figure 2:**
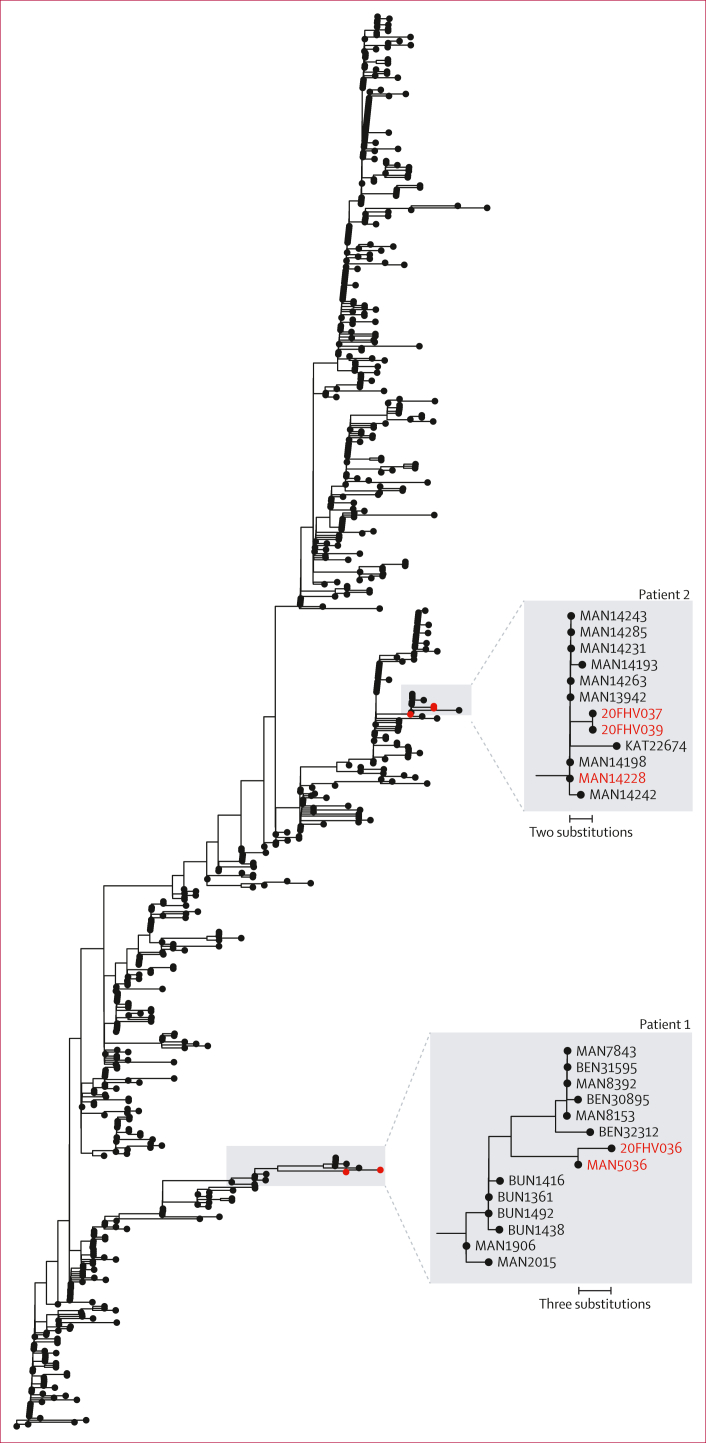
Maximum likelihood phylogenetic tree of the Ebola virus disease outbreak (2018–20) in Nord Kivu, Democratic Republic of the Congo A time-resolved phylogenetic tree was conducted with a Bayesian phylodynamic analysis of 677 genomes sequenced during this outbreak. Sequenced genomes from patients 1 and 2 during the first and second episodes of Ebola virus disease are highlighted in red.

We performed a Bayesian phylodynamic analysis to construct a time-resolved phylogenetic tree using 677 outbreak genomes from the North Kivu province (figure 2). The overall rate of evolution during the North Kivu outbreak was 0 ·90 × 10^–3^ substitutions per genomic site per year (95% highest posterior density interval [HPD] 0 ·81 × 10^–3^ – 0 ·99 × 10^–3^), matching previous analyses.^11^

For patient 1, the genome from the CSF sample (20FHV036) was phylogenetically closest to the genome from the first episode of Ebola virus disease, differing by only three nucleotide substitutions, despite 364 days having elapsed between the two episodes. The branch leading to 20FHV036 had a rate of 0·26 × 10^–3^ substitutions per genomic site per year (95% HPD 0·76 × 10^–^⁴ – 0·47 × 10^–3^), corresponding to a 3·5 times reduction in evolutionary rate.

For patient 2, the genomes from blood (20FHV039) and CSF (20FHV037) taken during the second episode were phylogenetically closest to a cluster of cases from January, 2020, including the genome from patient 2’s first episode (MAN14228), and diverged by only two nucleotide substitutions, despite 158 days having elapsed between the two episodes. The branch leading to 20FHV037 (CSF) had a rate of 0·37 × 10^–3^ substitutions per genomic site per year (95% HPD 0·89 × 10^–4^ – 0·72 × 10^–^³), corresponding to a 2·4 times reduction in evolutionary rate.

Patient 1’s substitutions occurred in non-coding regions. Patient 2’s substitutions produced one silent mutation in VP40 and another in the mucin-like domain of full-length GP. All five mutations occurred outside the glycan cap, GP1/2, and fusion loop full-length GP domains targeted by REG-EB3 anti-GP antibodies.^20^^,^^21^

From patient 1, anti-Ebola virus IgM antibodies and anti-Ebola virus GP IgG antibodies were detected starting from the first plasma sample tested (day 8 of symptom onset) throughout acute hospitalisation. Anti-Ebola virus NP IgG antibodies and anti-Ebola virus VP40 IgG antibodies were not detected initially, but increased from 8 days after symptom onset into the first episode. During relapse, anti-Ebola virus IgG (but not IgM) antibodies against NP, VP40, and GP were present in the blood sample. Only anti-Ebola virus NP IgG antibodies were detected in CSF during the relapse episode.

**Figure 3:**
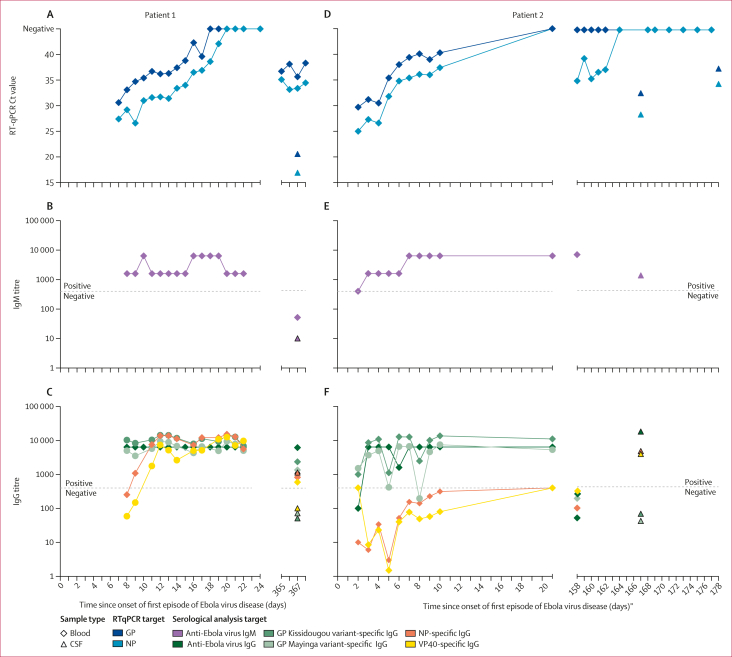
RT-qPCR and serological results for two patients with recrudescent Ebola virus disease over time Data on RT-qPCR Ct values, IgM titres, and IgG titres (including NP, GP [Kissidougou and Mayinga strains], and VP40 proteins) provided for patient 1 (A–C) and patient 2 (D–F) from the onset of the first episode of Ebola virus disease. Patient 1 received REGN-EB3 on day 7 after symptom onset in the first episode of disease, corresponding to 1 day before the first sample shown. Patient 2 received REGN-EB3 2 days after symptom onset in the first episode of disease. CSF=cerebrospinal fluid. Ct=cycle threshold. GP=glycoprotein. NP=nucleoprotein. qRT-PCR=quantitative RT-PCR. VP40=viral protein 40.

From patient 2, anti-Ebola virus IgM antibodies were detected in blood samples from day 3 throughout initial hospitalisation. Although anti-Ebola virus GP IgG antibodies were detected at high concentrations as early as day 4 of hospitalisation, anti-Ebola virus NP IgG antibodies and anti-Ebola virus VP40 IgG antibodies were never detected during initial hospitalisation. During relapse, anti-Ebola virus IgM antibodies were detected in blood samples, but anti-Ebola virus IgG antibodies to GP, NP, and VP40 were not detected. However, anti-Ebola virus NP IgG antibodies and anti-Ebola virus VP40 IgG antibodies were detected in CSF, along with anti-Ebola virus IgM antibodies (figure 3; appendix 2 pp 9–10).

## Discussion

During and in the aftermath of the Democratic Republic of the Congo’s tenth outbreak of Ebola virus disease, more than 1100 survivors have been regularly followed up by the national programme. Among these survivors, only three relapse events have been documented, suggesting that these events are rare but consequential. In this Article, we report the cases of two patients with fatal meningoencephalitis associated with persistence of Ebola virus. In patient 1, the virus persisted for 12 months after initial recovery from Ebola virus disease; in patient 2, it persisted for 5 months after initial recovery. Notably, these cases are the first such reports of relapse after vaccination with r-VSV-ZEBOV-GP and a now approved mAb treatment specific to Ebola virus. These contrasting cases provide the opportunity to consider the complex interaction between Ebola virus, the human host, and the characteristics of acute Ebola virus disease that could be potential risk factors for viral persistence in the CNS and associated meningoencephalitis. Current standards of care also merit consideration of whether and how receipt of vaccination with r-VSV-ZEBOV-GP or therapeutics specific to Ebola virus affect this risk. These observations prompt key research inquiry to inform best practices to prevent and treat these consequential events in the future.

Before the onset of the first episode of Ebola virus disease, both patients received vaccination with r-VSV-ZEBOV-GP following exposure to Ebola virus, according to the ring vaccination strategy applied in the Democratic Republic of the Congo in 2018–20.^22^ In both cases, initial presenting symptoms of the second episode were non-specific, and Ebola virus RNA was detected in blood samples at the onset of Ebola virus disease-like systemic illness for several consecutive days. Persistence of Ebola virus in the CNS was likely to be an initiating event for subsequent recrudescent disease (and certainly the ultimate cause of demise) in both patients; however, systemic manifestations and RT-qPCR positivity before, or at least simultaneous with, CNS manifestations imply the reverse is also possible. Although CSF was not sampled until later in the second episode, Ebola virus RNA was detectable in these samples for up to 19 days after the onset of relapse and at much higher amounts than in blood samples, similar to a previous case.^14^ Neither patient received mAb retreatment or remdesivir during the second episode, probably for several reasons. First, treatment was not immediately available in the remote locations where each patient received care. Second, both individuals were known survivors of Ebola virus disease with relatively low amounts of Ebola virus RNA in the blood. Third, in the setting of meningoencephalitis syndromes (with Ebola virus RNA detected in CSF) and with uncertainty about mAb distribution across the blood–brain barrier, the patients were not offered retreatment with REGN-EB3. Nonetheless, in the absence of more evidence, uncertainty around optimal management urges further research to establish the role of optimal supportive care specific to Ebola virus disease to improve patient outcomes.

The Ebola virus genomes from both patients were within the diversity of the Nord Kivu Ituri variant. The genomic analysis identified phylogenetic relatedness to each patient’s initial infection with Ebola virus, confirming in-host persistence of Ebola virus (likely in the CNS) rather than a new spillover event or reinfection from an undetected ongoing transmission chain or another survivor with persistent infection. In both patients, the Ebola virus genome from the second episode diverged at a slower evolutionary rate than was observed at the country-wide level, suggesting persistent infection with a low replication rate or latency. In the semen of male survivors of Ebola virus disease, viral replication has been shown to slow over time, suggesting persistence rather than latency;^23^ however, mechanisms of persistence might vary between immune-privileged sites. REGN-EB3 is a cocktail of three human IgG1 mAbs directed against different regions of the Ebola virus GP.^24^ Use of mAb treatments has been hypothesised to potentially lead to a generation of viral escape mutants.^19^ Importantly, inferred amino acid sequences from genomic analyses indicate that the GP regions from the relapse were probably not sufficiently modified to prevent recognition by the mAb cocktail and compromise therapeutic efficacy.

Comparison of patients showed interesting differences in host characteristics and the development of adaptive antibody immune responses specific to Ebola virus, at least as measured by binding antibodies (sample volume and laboratory setting limitations precluded neutralisation assays). Patient 1, an apparently healthy young woman, was vaccinated 5 days after presumptive exposure to Ebola virus (3 days before initial symptom onset) and received REGN-EB3 7 days after onset. She subsequently developed an antigen-specific humoral response with typical IgM and IgG antibody kinetics during acute illness, including detection of anti-GP, anti-NP, and anti-VP40 IgG antibodies in the plasma after acute illness and 1 year later.^25^^,^^26^ During and after acute Ebola virus disease, detection of anti-Ebola virus GP IgG antibodies cannot discriminate between GP-specific IgG antibodies resulting from vaccine-induced seropositivity, a response to Ebola virus infection, or a cross-detection of therapeutic mAbs targeted at the GP. Despite clinical recovery, the patient reported symptoms during convalescence that warranted consideration of potential neurological involvement that started during the first episode of disease. Unknown, but of great interest, is any contribution of her intervening pregnancy. Persistence of Ebola virus RNA has been detected in the breastmilk of pregnant survivors of Ebola virus disease; placental infection and transmission in utero have been described after subclinical Ebola virus infection in an unrecognised survivor.^27^^,^^28^ Pregnancy is recognised as a relative state of temporary immunosuppression; whether or how her pregnancy contributed to the initiation of recrudescent disease is unknown, but must be considered. The detection of anti-NP IgG antibodies in the CSF likely resulted from passive peripheral transfer, although intrathecal antibody synthesis cannot be ruled out.

Patient 2, a man aged 79 years, was vaccinated 1–9 days after probable exposure to Ebola virus, 4 days before symptom onset, and was treated with REGN-EB3 2 days later. Older age has been associated with the persistence of Ebola virus RNA in semen samples from cohorts from western Africa. The paucity of data limits generalisation to the CNS, but this factor might have contributed. Perhaps the most distinct difference to patient 1 is that a humoral immune response specific to Ebola virus after the development of anti-Ebola virus IgM antibodies during acute disease was never observed in patient 2. Anti-NP and anti-VP40 IgG antibodies were never detected in plasma samples during and after the first episode of Ebola virus disease, nor were anti-Ebola virus IgG antibodies to any antigen detected 5 months later. Validated detection of specific anti-VSV antibodies to assess humoral responses to the vaccine vector was not available. High concentrations of anti-GP IgG antibodies that were detected early in the first episode of the disease were likely secondary to cross-detection of therapeutic mAbs. The absence of anti-GP IgG antibodies both after initial illness and 5 months later indicates an absence of an appropriate response to both the vaccine antigen and subsequent infection with Ebola virus. Although impossible to causally substantiate, plausible contributors include the effect of corticosteroids during initial infection, unknown immunodeficiency or age-related impairment of humoral immune responses, and the potential effect of an unknown interaction between preventive or therapeutic interventions that might impair endogenous adaptive immune responses.

Both the r-VSV-ZEBOV-GP vaccine and therapeutic mAbs target the Ebola virus GP. Elapsed time between vaccination and REGN-EB3 treatment was 10 days for patient 1 and 6 days for patient 2. Studies in non-human primate models and data from the PALM clinical trial (albeit self-reported by patients who received the r-VSV-ZEBOV-GP vaccine) suggest that Ebola virus disease following vaccination might have reduced severity.^3^^,^^29^ How treatment with a mAb that targets the Ebola virus GP might interact with a developing response stimulated by the vaccine or Ebola virus GP and vice versa, is unknown, as is the interaction with endogenous humoral responses.^29^ Furthermore, although an appropriate clearance of viral RNAaemia was observed in both patients, we cannot be certain whether the cocktail of vaccination and treatment with REGN-EB3 modified or blunted the antibody response in these individuals and somehow impaired complete viral clearance, potentially leading to viral sequestration in immune-privileged sites.

During patient 2’s relapse, anti-Ebola virus IgM antibodies were detected in the periphery and in the CNS. Despite the absence of anti-Ebola virus IgG antibodies to any Ebola virus antigens in the blood, anti-Ebola virus IgG antibodies to all antigens were detected in CSF, suggesting the production of intrathecal antibodies. IgM is not known to passively diffuse across the blood–brain barrier; the character and kinetics of the immune response in the CNS suggest the presence of antigen-presenting cells and the activation of B and T lymphocytes specific to Ebola virus akin to a localised primary response. Data from non-human primate models suggest that CD68^+^ macrophages and microglia are a reservoir of persistent infection with Ebola virus in the CNS of animals who received treatment with mAbs.^19^ Further investigation is needed to define the cellular sites of persistent viral infection in the CNS of humans.

Both the vaccination and use of mAb therapeutics have improved the ability to control outbreaks of Ebola virus and reduce patient mortality. Our findings highlight the importance of long-term clinical, virological, and immunological monitoring of survivors of Ebola virus disease. Safe CSF sampling should be recommended in survivors who develop relevant neurological symptoms or signs. Evaluation of the peripheral biomarkers of Ebola virus persistence is greatly needed. These case studies highlight the need to explore strategies to prevent viral persistence in the CNS during acute Ebola virus disease, including investigating the role of combination therapeutics to target the virus (eg, pairing approved mAb therapeutics with small-molecule antivirals to improve penetration into the CNS) or host-targeted approaches (eg, endothelial stabilisation of the blood–brain barrier). Strategies to improve outcomes during meningoencephalitis episodes are also warranted, including optimised supportive care, the potential use of therapies specific to Ebola virus, and targeting CNS inflammation (eg, by cautious use of corticosteroids, depending on clinical presentation and with first-do-no-harm attention to the immune status of the patient). It is unknown whether a second course of one of the two licensed mAbs during relapse might confer additional benefit. Both remdesivir and high-dose corticosteroids were used for treatment in the only documented patient to survive meningoencephalitis associated with persistence of Ebola virus in the CNS to date; however, a causal benefit cannot be assumed.^14^ Key challenges for the care of the two patients in the present study were the rapid evolution of disease in a remote location and the fact that remdesivir was not immediately available. Preclinical and clinical studies are needed to further understand the interactions between the host, virus, and intervention that determine persistence of Ebola virus in the CNS and to assess the best preventive and therapeutic management strategies.

## Data sharing

The sequence data has been submitted to GenBank and genomes are available under accession numbers OQ348595 for 20FHV039, OQ348596 for 20FHV037, OQ348597 for 20FHV036, and OQ348598 for MAN5036. MT778640 (for MAN14228) and 677 genomes from the 2018–20 outbreak of Ebola virus disease have already been made available on Nextstrain, GitHub, and GenBank (accession numbers are listed in appendix 3). The Beauti xml file used for BEAST analysis is available on GitHub. Anonymised data will be made available upon reasonable request to the corresponding author.

## Declaration of interests

We declare no competing interests.
