# Supplementary material for: Fatal meningoencephalitis associated with Ebola virus persistence in two survivors of Ebola virus disease in the Democratic Republic of the Congo: a case report study
Source: Lancet Microbe. 2024 Oct;5(10):None. doi: 10.1016/S2666-5247(24)00137-X (PMC11464592; doi:10.1016/S2666-5247(24)00137-X)
Supplement: Supplementary appendix 1 [file mmc1.pdf]

THE LANCET
Microbe

Supplementary appendix 1

This translation in French was submitted by the authors and we reproduce it as supplied. It has not been peer reviewed. *The Lancet*'s editorial processes have only been applied to the original in English, which should serve as reference for this manuscript.

Cette traduction en français a été proposée par les auteurs et nous l'avons reproduite telle quelle. Elle n'a pas été examinée par des pairs. Les processus éditoriaux du *Lancet* n'ont été appliqués qu'à l'original en anglais et c'est cette version qui doit servir de référence pour ce manuscrit.

Supplement to: Mukadi-Bamuleka D, Edidi-Atani F, Morales-Betoulle ME, et al. Fatal meningoencephalitis associated with Ebola virus persistence in two survivors of Ebola virus disease in the Democratic Republic of the Congo: a case report study. *Lancet Microbe* 2024. https://doi.org/10.1016/S2666-5247(24)00137-X

Résumé

Antécédents :

Les sites de privilège immun, y compris les testicules et le système nerveux central, peuvent être des sources de persistance du virus Ebola (EBOV). Au cours de l'épidémie de maladie à virus Ebola (MVE) de 2018-2020 en République démocratique du Congo (RDC), des milliers de patients atteints de la MVE ont reçu, et ce pour la première fois, une vaccination et/ou un traitement par anticorps monoclonaux, ce qui a permis à un grand nombre de personnes de survivre à la maladie. Nous décrivons les caractéristiques cliniques, virologiques, génomiques virales et immunologiques de deux personnes guéries de la MVE, précédemment vaccinés et traités par anticorps monoclonaux qui, des mois après leur décharge initiale, ont développé un deuxième épisode de MVE qui s'est finalement compliqué d'une méningo-encéphalite mortelle associée à la persistance d'EBOV.

Méthodes :

Conformément aux protocoles de la réponse à la MVE et du programme de soin et de suivi des personnes guéries de la MVE, établis lors de l'épidémie de MVE de 2018-2020 en RDC, du sang, du plasma et du liquide céphalorachidien (LCR) ont été prélevés au cours des épisodes initiaux et secondaires de la MVE chez les deux cas reportés ici et ont été analysés par des techniques moléculaires (RT-qPCR, séquençage de nouvelle génération) et sérologiques (tests ELISA, Luminex).

Résultats :

Deux cas sont présentés, décrivant les épisodes initiaux et les rechutes de la MVE ainsi que le développement de méningo-encéphalite. Chez les deux patients, l'ARN d'EBOV a été détecté dans le sang et le LCR pendant l'épisode de rechute. Les génomes complets obtenus à partir d'échantillons de LCR du deuxième épisode ont révélé une parenté phylogénétique avec les génomes d'échantillons de sang de l'infection initiale, confirmant la persistance d'EBOV dans l'hôte. L'analyse sérologique a démontré une réponse humorale spécifique contre les antigènes d'EBOV, avec une cinétique IgM/IgG typique dans l'un des deux cas, mais l'incapacité à générer une réponse immunitaire adaptative endogène dans l'autre cas.

Interprétation :

Nous rapportons ici les deux premiers cas mortels de méningo-encéphalite associés à la persistance d'EBOV après avoir reçu la vaccination et un traitement anti-EBOV à base d'anticorps monoclonaux en RDC. Nos résultats soulignent l'importance d'un suivi à long terme des personnes guéries de la MVE, comprenant des aspects cliniques, virologiques et immunologiques, ainsi que le besoin urgent de nouvelles stratégies thérapeutiques pour prévenir et atténuer les conséquences individuelles et de santé publique de la persistance de l'EBOV.

Financement :

La présente étude a été financée par le ministère de la Santé de la RDC et l'Institut National de Recherche Biomédicale, avec l'aide du fonds de réserve pour la réponse rapide aux maladies infectieuses, les Centres pour le Control et Prevention des Maladies des États-Unis et l'Organisation Mondiale de la Santé, dans le cadre de la réponse à l'épidémie de maladie à virus Ebola. L'analyse Luminex a été réalisée dans le cadre de l'étude « Les vainqueurs d'Ebola » de l'Agence nationale de recherche sur le sida – Maladies infectieuses émergentes – de l'Institut National de la Santé et de la Recherche Médicale, de l'Institut National de Recherche pour le Développement et du Partenariat pour les essais cliniques entre l'Europe et les pays en développement.
