# Supplementary material for: Fatal meningoencephalitis associated with Ebola virus persistence in two survivors of Ebola virus disease in the Democratic Republic of the Congo: a case report study
Source: Lancet Microbe. 2024 Oct;5(10):None. doi: 10.1016/S2666-5247(24)00137-X (PMC11464592; doi:10.1016/S2666-5247(24)00137-X)
Supplement: Supplementary appendix 2 [file mmc2.pdf]

THE LANCET
Microbe

Supplementary appendix 2

This appendix formed part of the original submission and has been peer reviewed.
We post it as supplied by the authors.

Supplement to: Mukadi-Bamuleka D, Edidi-Atani F, Morales-Betoulle ME, et al. Fatal meningoencephalitis associated with Ebola virus persistence in two survivors of Ebola virus disease in the Democratic Republic of the Congo: a case report study. *Lancet Microbe* 2024. https://doi.org/10.1016/S2666-5247(24)00137-X

Supplementary Appendix

This appendix has been provided by the authors to give readers additional information about the work presented in the main publication by Daniel Mukadi-Bamuleka, François Edidi-Atani, Maria E. Morales-Betoulle, Anaïs Legand, Antoine Nkuba-Ndaye, Junior Bulabula-Penge, Placide Mbala-Kingebeni, et al., titled "**Fatal meningoencephalitis associated with EBOV persistence in two survivors in the Democratic Republic of the Congo: a case report study**"

Table of Contents

Supplementary Appendix 1

Table of Contents 2

1 Supplemental Materials and Methods 3

1.1 General information on vaccination strategies and EVD patient and survivor care during the 2018–2020 outbreak in DRC 3

1.2 Next-generation sequencing and genomic analyses 4

1.3 Serological testing for anti-EBOV antibodies using CDC's in-house ELISA 5

1.3.1 Specimen inactivation 5

1.3.2 CDC IgM and IgG ELISA 5

1.3.3 Luminex serology 6

2 Supplemental information on timelines from exposure to relapse and laboratory testing results of specimens collected during 1st and 2nd EVD episodes for each case 8

2.1 Table S1. Time intervals between initial exposure to EBOV, vaccination, admission to ETU, REGN-EB3 treatment, discharge, and relapse for each case. 8

2.2 Table S2. RT-qPCR, sequencing, and serology results for Case 1 during initial EVD episode and relapse. 9

2.3 Table S3. RT-qPCR, sequencing, and serology results for Case 2 during initial EVD episode and relapse. 10

3 Additional clinical laboratory results during relapse for both cases 11

3.1 Additional laboratory results for Case 1 during EVD relapse 11

Figure S1. Rapid diagnostic test results for Case 1 11

3.2 Additional laboratory results for Case 1 during EVD relapse, June 2020 12

Table S4. Biological parameter follow-up of Case 1 12

3.3 Additional laboratory results for Case 2 during EVD relapse, June-July, 2020 13

Table S5. Biological parameter follow-up during hospitalization of Case 2. 13

4 References for Supplementary Appendix 14

1 Supplemental Materials and Methods

1.1 General information on vaccination strategies and EVD patient and survivor care during the 2018–2020 outbreak in DRC

During the 2018–2020 Ebola virus disease (EVD) outbreak in DRC, vaccination against EVD with the rVSV-ZEBOV-GP vaccine was implemented following recommendations of the Strategic Advisory Group of Experts (SAGE) on immunization in 2017 and 2019.[1,2] In general, a ring or geographic vaccination strategy was used for individuals considered contacts, or contacts of contacts. Vaccination status of the two cases reported here was recorded based on information reported by the patients upon admission to the Ebola Treatment Unit (ETU), or on information available in the various clinical and epidemiological databases. Once admitted to the ETU, EVD patients were managed per WHO recommendations on optimized supportive care for EVD.[3] Some patients were enrolled in a randomized controlled trial (PALM study) and received therapeutics such as the REGN-EB3 that was provided to the two cases described here.[4] Since 2018, the DRC Ministry of Health and the National Institute for Biomedical Research (INRB), with support from WHO and partners, implemented an EVD survivor care program that offered monthly consultations to all EVD survivors, including clinical and psychological care and RT-PCR testing of semen, vaginal fluids, and breast milk. Antenatal care was provided to women like Patient 1, who became pregnant after EVD recovery. A referral system to a higher level of care was organized for complications that could not be managed at the clinic. In case of any emergencies, referrals and hospital admissions were supported by the program. The program was made available to all EVD survivors upon discharge from the ETU, following WHO guidelines.[5]

1.2 Next-generation sequencing and genomic analyses

We sequenced blood and cerebrospinal fluid (CSF) samples collected during the relapse episode in 2020, from both patients (Case 1 20FHV036, 20FHV038; Case 2 20FHV037, 20FHV039), along with a sample taken during the initial EVD episode of Case 1 (MAN5036) for comparison. We had previously sequenced a sample from the initial EVD episode in Case 2 (MAN14228) in early 2020 while providing genomic epidemiology support during the 10th EVD outbreak. Samples were extracted using the Qiagen Viral RNA Mini kit (Qiagen, Valencia, CA, USA). MAN5036 and MAN14228 were sequenced using a targeted enrichment technique on the Illumina platform (Illumina Inc., San Diego, CA, USA) as previously described in the Supplement of Mbala-Kingebeni et al.[6] Samples collected during the relapse episode, were sequenced using an amplicon-based approach on the Illumina platform as previously described in the Supplement of Mbala-Kingebeni *et al*.[7] Sequences are available in GenBank under accession numbers: MT778640, and OQ348595-OQ348598.

A Bayesian phylodynamic analysis was performed to construct a time-resolved phylogeny using samples MAN14228, MAN5036, 20FHV036, and 20FHV037, along with 677 other North Kivu outbreak genomes. First, multiple sequence alignment was performed with MAFFT, maximum likelihood tree inferred using IQ-TREE, and tree visualized with TempEst v1.5.3 to assess clock-likeness and absence of outliers (excluding known and suspected persistent infection-related cases).[8,9] Time-calibrated phylogenies were estimated with the MCMC method implemented in BEAST v1.10.4.[10] We used an HKY+G nucleotide substitution model, with a local clock model and a non-informative continuous time Markov chain reference prior on the molecular clock rate with a separate prior for sets of cases known or suspected to be related to persistent infections, and a Skygrid coalescent prior. The analysis was run for 80 million chains, sampling every 8,000. Tracer v1.7.1 was used to assess run convergence (i.e., effective sample size > 200).[11] The

sequence fasta and BEAST XML files are available at https://github.com/cathrnbp/paper-2023-ebolacsf.

1.3 Serological testing for anti-EBOV antibodies using CDC's in-house ELISA

1.3.1 Specimen inactivation

Blood and CSF specimens were collected from both Case 1 and Case 2 at multiple different time points. Prior to serology testing, specimens were inactivated in a Class III biosafety cabinet (Cleatech HEPA Filtered Glove box, Cleatech LLC, https://www.cleatech.com). For inactivation, 42 µL of blood were diluted in 1 mL of Master Plate buffer containing 5% skim milk (Fischer Sci, Pittsburg, PA, USA) in PBS (Sigma-Aldrich, St. Louis, MO, USA), 0·5% Triton X100 (Sigma-Aldrich, St. Louis, MO, USA), and 0·5% Tween-20 (Sigma-Aldrich, St. Louis, MO, USA). For CSF samples, 50 µL of specimen were diluted in 200 µL of Master Plate diluent. Tubes were then placed in a heat block (Fischer Sci, Pittsburg, PA, USA) at 56°C for 15 min, mixed, and incubated 30 min at 56°C. The samples were then tested in a biosafety level 2 (BSL-2) laboratory using the CDC IgM and IgG ELISA described below.

1.3.2 CDC IgM and IgG ELISA

The methods for in-house IgM and IgG ELISAs were previously described.[12,13] Viral antigens were prepared with EBOV-Mayinga strain (spp. *Orthoebolavirus zairense*) in Vero-E6 cells (CDC, Atlanta, GA, USA) in a CDC BSL-4 laboratory. Infected cells were processed by detergent basic buffer extraction for lysate antigen preparations (IgG) and by cell suspension in 0·1 M Tris (pH 8·5), followed by a freeze and thaw cycle to produce a cell slurry (IgM); cell lysate and cell slurry were then inactivated by gamma-irradiation as described.[13,14] Mock control antigen (uninfected Vero-E6 cells) lysate (for IgG) and cell slurry (for IgM) were prepared in a

similar manner. Inactivated specimens were tested in a BSL-2 laboratory by 4-fold serial dilutions from 1:100 (blood) or 1:20 (CSF) to 1:64000. To detect human IgG, anti-human IgG horseradish peroxidase conjugate (Accurate Chemical, Westbury, NY, USA) and H_2O_2-ABTS substrate system (KPL, Sera Care, Milford, MA, USA) were used. For IgG, an adjusted OD (difference between values obtained with positive samples and negative controls) of > 0·2 was required for each dilution to be considered positive and for a titer to be assigned accordingly. Specimens were considered positive if the titer was ≥1:400 (for blood) or ≥1:80 (for CSF) and the sum of the adjusted OD was ≥0·95. For IgM ELISA, anti-human IgM antibody (KPL, Sera Care, Milford, MA, USA) was used to capture IgM antibodies from the serially diluted samples, followed by EBOV or mock antigens. Captured antigens were detected using a polyclonal anti-EBOV rabbit antibody produced at CDC, followed by an anti-rabbit horseradish peroxidase conjugate (BioRad, Hercules, California, USA) and the H_2O_2-ABTS (Sera Care, Milford, MA, USA) substrate system. For IgM, adjusted OD > 0·1 was required for each dilution to be considered positive and for a titer to be assigned accordingly. Specimens were considered positive if the titer was ≥1:400 (for blood) or ≥1:80 (for CSF) and the sum of the adjusted OD was ≥0·45. Known positive and negative controls were ran in parallel to samples for each ELISA test to ensure adequate performance of the assays.

1.3.3 Luminex serology

Plasma and CSF samples from the two cases were transferred to the INRB biobank and stored at -80°C until laboratory analyses were performed. The Luminex assay was performed using four commercially available recombinant viral proteins from EBOV (spp. *Orthoebolavirus zairense)* representing EBOV glycoprotein (Kissidougou strain 2014 glycoproteins [GP-EBOV-k] and Mayinga strain 1976 [GP-EBOV-m]; Sino Biological, Beijing, China), EBOV nucleoprotein (Kissidougou strain 2014 nucleoproteins [NP-EBOV]; Sino Biological]) and EBOV VP-40

(Kissidougou strain 2014 40-kDa viral protein [VP-EBOV; Sino Biological] to detect anti-Ebola IgG on the x.MAP Technology (Luminex, Austin, TX, USA). Briefly, plasma samples were diluted 1:1000 in dilution buffer, composed of deionized distilled water, 1% bovine serum albumin, 5% fetal bovine serum, 1% hypertonic phosphate buffered saline, and 0.2% Tween-20. After mixing the samples with beads previously coupled with a specific antigen, the plates were incubated overnight at 4°C. CSF samples were diluted 1:500 in dilution buffer and similarly treated. Prior to adding the secondary antibody and a fluorescent protein (streptavidin phycoerythrin) successively, the plates were washed five times. The plates were then read using Magpix. Results were expressed in median fluorescence intensity (MFI), which assumes at least 100 beads read per analyte. For each antigen, reported sensitivity and specificity were significantly above 90%.[10,11] We used the following thresholds to determine seropositivity: 400 MFI for GP (Mayinga strain), 600 MFI for NP, and 650 MFI for VP40. These thresholds were previously established in various studies that assessed survivors, their contacts, and a large panel of EBOV-negative individuals.[15,16] Based on the results of the serological tests, patients were considered seropositive when their sample were reactive to at least two of the three EBOV antigens.

2 Supplemental information on timelines from exposure to relapse and laboratory testing results of specimens collected during 1st and 2nd EVD episodes for each case

2.1 Table S1. Time intervals between initial exposure to EBOV, vaccination, admission to ETU, REGN-EB3 treatment, discharge, and relapse for each case.

Time between:	Case 1	Case 2
EBOV exposure[1] → rVSV-ZEBOV-GP vaccination[2]	5 days	1–9 days
EBOV exposure → EVD symptom onset	8 days	5–14 days
EBOV exposure → admission to ETU	14 days	7–16 days
EBOV exposure → treatment with REGN-EB3	15 days	7–16 days
rVSV-ZEBOV-GP vaccination → EVD symptom onset	3 days	4 days
rVSV-ZEBOV-GP vaccination → treatment with REGN-EB3	10 days	6 days
EVD symptom onset → treatment with REGN-EB3	7 days	2 days
rVSV-ZEBOV-GP vaccination → ETU discharge (1st episode)	25 days	25 days
Treatment with REGN-EB3 → ETU discharge (1st episode)	15 days	19 days
First discharge → EVD relapse	342 days	137 days

[1]The exact day of exposure could only be estimated based on the information available in the DRC epidemiology or ETU databases, or case investigation records. For Case 1, exposure was assumed to have occurred at the communal clinic she visited in June, on the same day when two confirmed fatal EVD cases were reportedly present in that health care facility. For Case 2, the period of exposure was estimated based on his records of caring for and assisting to the funeral of a family member who was a confirmed EVD case.

[2]The rVSV-ZEBOV vaccination record information date for each case was obtained from the DRC epidemiology databases or ETU or case investigation records.

2.2 Table S2. RT-qPCR, sequencing, and serology results for Case 1 during initial EVD episode and relapse.

Date of onset of 1st episode was in June, 2019, and 2nd episode was in June, 2020. Discharge after 1st episode was in July, 2019, and date of death after 2nd episode was June, 2020. Patient received REGN-EB3* in July, 2019, one day before collection of first sample tested by serological assays. At relapse, blood samples were negative for anti-EBOV IgM and positive for anti-EBOV IgG; CSF samples were negative for IgM and positive for IgG. nd: not done; CSF: cerebrospinal fluid; Kiss: Kissidougou EBOV variant; May: Mayinga EBOV variant.

Sample type	Lab ID	Days since first EVD onset	Days since second EVD onset	GeneXpert RT-qPCR (Ct)		Genome coverage (%)	CDC Serology Assay				Luminex Serology Assay IgG Titers and Result				
				GP	NP		anti-Ebola virus IgM Titer	anti-Ebola virus IgM Result	anti-Ebola virus IgG Titer	anti-Ebola virus IgG Result	anti-NP	anti-GP_{Kiss}	anti-GP_{May}	anti-VP40	Result
**First episode of EVD (symtom onset, June 2019)**															
Blood	MAN 5036	7		30.6	27.4	99.9	nd	nd	nd	nd	nd	nd	nd	nd	nd
Plasma	MAN 5112	8		33.1	29.2	nd	1600	Positive	6400	Positive	254	10308	5025	59	Negative
Plasma	MAN 5153	9		34.7	26.6	nd	1600	Positive	6400	Positive	1089	8383	3536	150	Positive
Plasma	MAN 5203	10		35.4	31.0	nd	6400	Positive	6400	Positive	5967	12117	6784	802	Positive
Plasma	MAN 5250	11		36.7	31.6	nd	1600	Positive	6400	Positive	7584	10432	5758	1779	Positive
Plasma	MAN 5302	12		36.2	31.7	nd	1600	Positive	6400	Positive	13816	14323	9448	7386	Positive
Plasma	MAN 5356	13		36.3	31.4	nd	1600	Positive	6400	Positive	13816	14255	8932	5192	Positive
Plasma	MAN 5395	14		37.4	33.4	nd	1600	Positive	6400	Positive	11294	11786	6840	2636	Positive
Plasma	MAN 5445	15		38.8	34.0	nd	1600	Positive	6400	Positive	9354	12305	7209	4390	Positive
Plasma	MAN 5495	16		42.3	36.5	nd	6400	Positive	6400	Positive	7318	7977	4303	5032	Positive
Plasma	MAN 5528	17		39.6	36.9	nd	6400	Positive	6400	Positive	12162	11287	6647	5141	Positive
Plasma	MAN 5576	18		0.0	38.6	nd	6400	Positive	6400	Positive	574	5374	2156	546	Positive
Plasma	MAN 5658	19		0.0	42.1	nd	6400	Positive	6400	Positive	12160	9324	4975	10870	Positive
Plasma	MAN 5695	20		0.0	0.0	nd	1600	Positive	6400	Positive	15272	14111	9443	12645	Positive
Plasma	MAN 5778	21		0.0	0.0	nd	1600	Positive	6400	Positive	12882	12791	7939	7147	Positive
Blood	MAN 5820	22		0.0	0.0	nd	1600	Positive	6400	Positive	5633	6951	5019	9721	Positive
Blood	MAN 5953	24		0.0	0.0	nd	nd	nd	nd	nd	nd	nd	nd	nd	nd
**Second episode of EVD (symptom onset, Jun 2020)**															
Blood	none	365	1	35.1	33.6	nd	nd	nd	nd	nd	nd	nd	nd	nd	nd
Blood	none	366	2	36.4	31.8	nd	nd	nd	nd	nd	nd	nd	nd	nd	nd
Blood	20FHV038	367	3	34.1	32	27.5	50	Negative	6400	Positive	841	2432	1389	603	Positive
CSF	20FHV036	367	3	20.1	16.7	99.5	10	Negative	1280	Positive	1170	51	72	101	Negative
Blood	none	368	4	36.6	33	nd	nd	nd	nd	nd	nd	nd	nd	nd	nd

*Note: the antibodies contained in REGN-EB3 cross react with the CDC's IgG ELISA and, if present, would give a positive signal (data not shown); nd = test not done

2.3 Table S3. RT-qPCR, sequencing, and serology results for Case 2 during initial EVD episode and relapse.

Date of onset of 1st episode was January, 2020, and 2nd episode was June, 2020. Date of discharge after 1st episode was February 2020, and date of death after 2nd episode was July, 2020. Patient received REGN-EB3* in January, after the first sample collection. At relapse, blood samples were positive for IgM and negative for IgG; CSF was positive for IgM and IgG. nd: not done; CSF: cerebrospinal fluid; Kiss: Kissidougou EBOV variant; May: Mayinga EBOV variant.

Sample type	Lab ID	Days since first EVD onset	Days since second EVD onset	GeneXpert RT-PCR (Ct)		Genome coverage (%)	CDC Serology Assay				Luminex Serology Assay IgG Titers and Result				
				GP	NP		anti-Ebola virus IgM Titer	anti-Ebola virus IgM Result	anti-Ebola virus IgG Titer	anti-Ebola virus IgG Result	anti-NP	anti-GP_{Kiss}	anti-GP_{May}	anti-VP40	Result
**First episode of EVD (symtom onset, January 2020)**															
Plasma	MAN 14228	2		29.7	25.0	99.7	400	Positive	100	Negative	10	995	1520	403	Negative
Plasma	MAN 14255	3		31.2	27.3	nd	1600	Positive	6400	Positive	6	8580	3690	9	Negative
Plasma	MAN 14298	4		30.5	26.6	nd	1600	Positive	6400	Positive	34	10841	4933	23	Negative
Plasma	MAN 14370	5		35.4	31.8	nd	1600	Positive	6400	Positive	3	1118	417	2	Negative
Plasma	MAN 14413	6		38.0	34.8	nd	1600	Positive	1600	Positive	52	12763	6571	40	Negative
Plasma	MAN 14450	7		39.4	35.4	nd	6400	Positive	6400	Positive	155	12645	6698	78	Negative
Plasma	MAN 14505	8		40.1	36.1	nd	6400	Positive	6400	Positive	141	2479	196	49	Negative
Plasma	MAN 14550	9		39.0	36.0	nd	6400	Positive	6400	Positive	227	10081	4595	58	Negative
Plasma	MAN 14599	10		40.3	37.4	nd	6400	Positive	6400	Positive	313	13609	7484	80	Negative
Blood	none	21		0.0	0.0	nd	6400	Positive	6400	Positive	400	11039	5320	400	Negative
**Second episode of EVD (symptom onset, Jun 2020)**															
Blood	20FHV039	158	1	0.0	35.0	97.1	6400	Positive	50	Negative	97	252	185	302	Negative
Blood	none	159	2	0.0	39.4	nd	nd	nd	nd	nd	nd	nd	nd	nd	nd
Blood	none	160	3	0.0	35.4	nd	nd	nd	nd	nd	nd	nd	nd	nd	nd
Blood	none	161	4	0.0	36.7	nd	nd	nd	nd	nd	nd	nd	nd	nd	nd
Blood	none	162	5	0.0	37.2	nd	nd	nd	nd	nd	nd	nd	nd	nd	nd
Blood	none	164	7	0.0	0.0	nd	nd	nd	nd	nd	nd	nd	nd	nd	nd
CSF	20FHV037	167	10	32.6	28.4	99.7	1280	Positive	16000	Positive	4339	66	41	3576	Positive
Blood	none	168	11	0.0	0.0	nd	nd	nd	nd	nd	nd	nd	nd	nd	nd
Blood	none	169	12	0.0	0.0	nd	nd	nd	nd	nd	nd	nd	nd	nd	nd
Blood	none	171	14	0.0	0.0	nd	nd	nd	nd	nd	nd	nd	nd	nd	nd
Blood	none	173	16	0.0	0.0	nd	nd	nd	nd	nd	nd	nd	nd	nd	nd
Blood	none	175	18	0.0	0.0	nd	nd	nd	nd	nd	nd	nd	nd	nd	nd
Blood	none	177	20	0.0	0.0	nd	nd	nd	nd	nd	nd	nd	nd	nd	nd
CSF	none	178	21	37.4	34.4	nd	nd	nd	nd	nd	nd	nd	nd	nd	nd

*Note: the antibodies contained in REGN-EB3 cross react with the CDC's IgG ELISA and, if present, would give a positive signal (data not shown); nd = test not done.

3 Additional clinical laboratory results during relapse for both cases

3.1 Additional laboratory results for Case 1 during EVD relapse

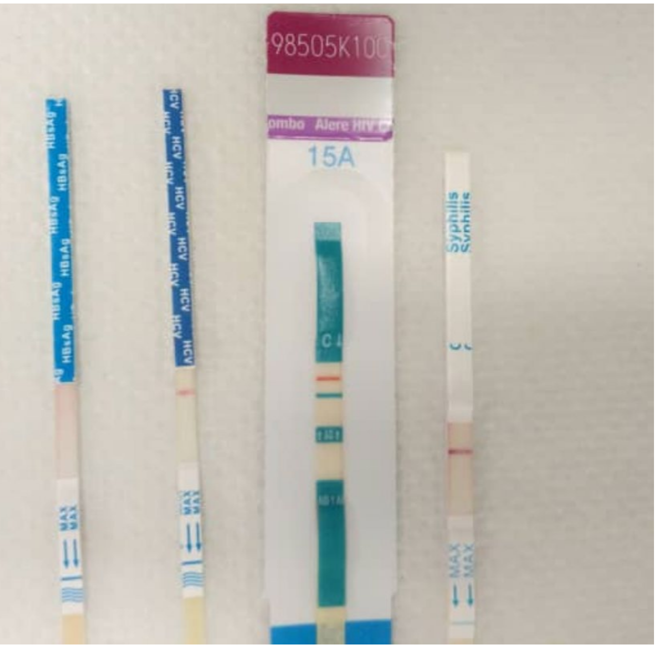

Figure S1. Rapid diagnostic test results for Case 1. All results are negative. From left to right: hepatitis B virus surface antigen, hepatitis C virus antibody, HIV antigen and antibody, and syphilis rapid tests.

3.2 Additional laboratory results for Case 1 during EVD relapse, June, 2020

Table S4. Biological parameter follow-up of Case 1

Parameter*	Units	Reference range	Day 1 post EVD relapse	Day 2 post EVD relapse	Day 3 post EVD relapse	Day 4 post EVD relapse0
**Blood chemistry (Piccolo-Metlac 12)**						
Glu	mg/dL	73–113	151	133	-	126
BUN	mg/dL	7–22	7	6	-	6
CRE	mg/dL	0·6–1·2	0·5	0·2	-	0·2
Na+	mmol/L	138–145	147	133	-	138
K+	mmol/L	3·6–5·1		3·1	-	2·3
CL-	mmol/L	98–108	98	100	-	99
Tco2	mmol/L	18–33	16	21	-	21
Ca	mg/dL	8–10·3	<8	8·6	-	9·1
PHOS	mg/dL	2·2–4·1	3·4	2·3	-	3·4
Mg	mg/dL	1·6–2·3	0·7	1·7	-	1·8
Alb	g/dL	3·3–5·5	2·8	2·8	-	2·6
LAC	mm/L	0·53–2·1	4·8	1·8	-	1·3
**Hematology (Sysmex)**						
WBC	$10^3/\mu L$	3–15	15·2	18·9	-	19
RBC	$10^6/\mu L$	2·5–5·5	3.5	3.3	-	3
HGB	g/L	8–17	10·1	9·3	-	8·6
HCT	%	26–50	31	28·5	-	25·4
MCV	/fL	86–110	87·8	85·1	-	84·4
MCH	/pg	26–38	28·6	27·8	-	28·6
MCHC	g/dL	31–37	32·6	32·6	-	33·9
PLT	$10^3/\mu L$	150–400	450	459	-	363
LYM	%	5–55	10·5	9·3	-	6·5
MXB	%	1–20	4·2	4·8	-	4
NEUT M	%	45–95	85·3	85·9	-	89·5
LYM	$10^3/\mu L$	-	1·6	1·8	-	1·2
MXD	$10^3/\mu L$	-	0·6	0·9	-	0·8
NEUT	$10^3/\mu L$	-	13	16·2	-	17
RDW-SD	/fL	37–54	62·2	56·2	-	54·5
RDW-CV	%	11–16	20·4	19	-	18

* Piccolo, and Sysmex pocH-110

3.3 Additional laboratory results for Case 2 during EVD relapse, June-July, 2020

Table S5. Biological parameter follow-up during hospitalization of Case 2.

			June 2020 (Days post second EVD onset)									July 2020				
**Parameter***	**Units**	**Reference range**	**D1**	**D2**	**D3**	**D4**	**D5**	**D6**	**D7**	**D8**	**D10**	**D12**	**D14**	**D16**	**D17**	**D18**
**Blood chemistry (Piccolo-Amylte13/Metlac 12)**																
Glu	mg/dL	73–113	138	131	120	101	110	-	171	122	109	119	156	204	-	212
BUN	mg/dL	7–22	18	21	25	21	17	-	20	19	20	18	13	17	-	-
CRE	mg/dL	0·6–1·2	1·3	1·9	1·5	1·5	1·3	-	0·6	1·5	1·1	1·3	0·7	0·7		1·1
TBIL	mg/dL	0·2–1·6	0·6	0·7	0·4	-	0·5	-	-	-	-	-	-	-	-	0·4
Alb	g/dL	3·3–5·5	2·3	3·1	2·7	2·8	2·6	-	2·6	2·9	3	2·5	2·4	2·4	-	2·4
ALT	U/L	10–47	8	11	12	-	12	-	-	-	-	-	-	-	-	46
AST	U/L	11–38	28	28	25	-	22	-	-	-	-	-	-	-	-	34
CK	U/L	30–380	400	361	222	-	76	-	-	-	-	-	-	-	-	-
AMY	U/L	14–97	61	68	112	-	74	-	-	--	-	-	-	-	-	69
Na+	mmol/L	138–145	170	138	137	132	133	-	119	121	119	119		114	-	117
K+	mmol/L	3·6–5·1	8·5	3·8	3·7	4·6	4·	-	4·3	4·9	3·9	3·9	4·9	4·8	-	5·8
CL-	mmol/L	98–108	-	-	-	100	-	-	88	87	85	88	82	81	-	-
Tco2	mmol/L	18–33	-	-	-	30	-	-	20	20	21	22	20	22	-	-
Ca	mg/dL	8–10·3	4	9	9	8·7	8·6	-	8·4	8·6	8·3	8	7·8	8·3	-	9·5
PHOS	mg/dL	2·2–4·1	-	-	-	4	-	-	3·8	3·2	4·7	3·1	2·7	2·8	-	-
Mg	mg/dL	1·6–2·3	-	-	-	1·7	-	-	1·8	1·8	1·8	1·6	1·6	1·7	-	-
LAC	mm/L	0·5–2·1	-	-	-	1·7	-	-	4·2	3·9	1·5	1·8	2·8	3·6	-	-
CRP	mg/L	≤7·5	22·2	34·6	19·8	-	7·5	-	-	-	-	-	-	-	-	>200
**Hematology (Sysmex)**																
WBC	10^3/µL	3–15	9·	7·9	7·6	8·1	6·2	-	-	-	10·7	4·3	3·8	5·2	-	9·4
RBC	10^6/µL	2·5–5·5	3·2	3·7	3·7	2·7	3·7	-	-	-	3·8	3·2	3·4	3·7	-	4·1
HGB	g/L	8–17	9·3	10·8	10·7	10·7	10·7	-	-	-	11·2	9·4	10·4	10·5	-	11·5
HCT	%	26–50	30	31·8	31·3	31·1	30·8	-	-	-	30·5	26·3	27·8	29·7	-	32·7
MCV	/fL	86–110	93·5	85·9	84·6	84·5	83	-	-	-	80·3	81·7	80·6	80·3	-	80
MCH	/pg	26–38	29	29·2	28·9	29·1	28	-	-	-	29·5	29·2	30·1	28·4	-	28·1
MCHC	g/dL	31–37	31	34	34·2	34·4	34	-	-	-	36·7	37·5	37·4	35·4	-	35·2
PLT	10^3/µL	50–400	73	161	160	191	172	-	-	-	-	265	-	188	-	235
LYM	%	5–55	10·8	22·9	20·3	17·4	23	-	-	-	13·4	10·8	18·2	7·6	-	7·7
MXB	%	1—20	10.0	14·5	7·4	4·9	7·3	-	-	-	6·8	7·5	3·5	3·9	-	1·8
NEUT M	%	45–95	79·2	62·6	72·3	77·7	69·2	-	-	-	79·8	81·7	78·3	88·5	-	90·5
LYM	10^3/µL	-	1	1·8	1·5	1·4	1·5	-	-	-	1·4	0·5	0·7	0·4	-	0·7
MXD	10^3/µL	-	0·9	1·1	0·6	0·4	0·5	-	-	-	0·7	0·3	0·1	0·2	-	0·2
NEUT	10^3/µL	-	7·1	5	5·5	6·3	4·2	-	-	-	8·6	3·5	3	4·6	-	8·5
RDW-SD	/fL	37–54	53·5	43·2	43·1	42	40·2	-	-	-	39·1	40·4	39·4	40·4	-	41·1
RDW-CV	%	11–16	16·2	14·2	14·2	13·8	13·5	-	-	-	13·8	14	14·1	14·3	-	14·5
MPV	/fL	9–13	13·8	11	11·3	11·3	12·9	-	-	-	-	-	-	0·2	-	9·7

* Piccolo, and Sysmex pocH-110

4 References for Supplementary Appendix

1. WHO. Preliminary results on the efficacy of rVSV-ZEBOV-GP Ebola vaccine using the ring vaccination strategy in the control of an Ebola outbreak in the Democratic Republic of the Congo: an example of integration of research into epidemic response. 2019. https://www.who.int/publications/m/item/preliminary-results-on-the-efficacy-of-rvsv-zebov-gp-ebola-vaccine-using-the-strategy-in-the-control-of-an-ebola-outbreak (accessed 09/09/2022).

2. Wong G, Qiu X, Bi Y, et al. More challenges from Ebola: infection of the central nervous system. *J Infect Dis* 2016; **214**(suppl 3): S294-S6.

3. WHO. WHO_Optimized supportive care for Ebola virus disease_2019.pdf. 2019. https://www.who.int/publications/i/item/optimized-supportive-care-for-ebola-virus-disease (accessed 09/09/2022).

4. Mulangu S, Dodd LE, Davey RT, et al. A randomized, controlled trial of Ebola virus disease therapeutics. *N Engl J Med* 2019; **381**(24): 2293-303.

5. WHO. Interim Guidance: Clinical care for survivors of Ebola virus disease, 2016.

6. Mbala-Kingebeni P, Aziza A, Di Paola N, et al. Medical countermeasures during the 2018 Ebola virus disease outbreak in the North Kivu and Ituri Provinces of the Democratic Republic of the Congo: a rapid genomic assessment. *Lancet Infect Dis* 2019; **19**(6): 648-57.

7. Mbala-Kingebeni P, Pratt C, Mutafali-Ruffin M, et al. Ebola virus transmission initiated by relapse of systemic Ebola virus disease. *N Engl J Med* 2021; **384**(13): 1240-7.

8. Katoh K, Kuma K, Toh H, Miyata T. MAFFT version 5: improvement in accuracy of multiple sequence alignment. *Nucleic Acids Res* 2005; **33**(2): 511-8.

9. Nguyen LT, Schmidt HA, von Haeseler A, Minh BQ. IQ-TREE: a fast and effective stochastic algorithm for estimating maximum-likelihood phylogenies. *Mol Biol Evol* 2015; **32**(1): 268-74.

10. Suchard MA, Lemey P, Baele G, Ayres DL, Drummond AJ, Rambaut A. Bayesian phylogenetic and phylodynamic data integration using BEAST 1.10. *Virus Evol* 2018; **4**(1): vey016.

11. Rambaut A, Drummond AJ, Xie D, Baele G, Suchard MA. Posterior summarization in Bayesian phylogenetics using Tracer 1.7. *Syst Biol* 2018; **67**(5): 901-4.

12. Ksiazek TG, Rollin PE, Williams AJ, et al. Clinical virology of Ebola hemorrhagic fever (EHF): virus, virus antigen, and IgG and IgM antibody findings among EHF patients in Kikwit, Democratic Republic of the Congo, 1995. *J Infect Dis* 1999; **179 Suppl 1**: S177-87.

13. Ksiazek TG, West CP, Rollin PE, Jahrling PB, Peters CJ. ELISA for the detection of antibodies to Ebola viruses. *J Infect Dis* 1999; **179 Suppl 1**: S192-8.

14. Bausch DG, Rollin PE, Demby AH, et al. Diagnosis and clinical virology of Lassa fever as evaluated by enzyme-linked immunosorbent assay, indirect fluorescent-antibody test, and virus isolation. *J Clin Microbiol* 2000; **38**(7): 2670-7.

15. Diallo MSK, Rabilloud M, Ayouba A, et al. Prevalence of infection among asymptomatic and paucisymptomatic contact persons exposed to Ebola virus in Guinea: a retrospective, cross-sectional observational study. *Lancet Infect Dis* 2019; **19**(3): 308-16.
16. Diallo MSK, Ayouba A, Keita AK, et al. Temporal evolution of the humoral antibody response after Ebola virus disease in Guinea: a 60-month observational prospective cohort study. *Lancet Microbe* 2021; **2**(12): e676-e84.
